# Use of airway clearance therapy in children hospitalised with acute lower respiratory tract infections in a South African paediatric hospital

**DOI:** 10.4102/sajp.v76i1.1367

**Published:** 2020-02-19

**Authors:** Lieselotte Corten, Brenda M. Morrow

**Affiliations:** 1 Department of Physiotherapy, University of Brighton, Eastbourne, United Kingdom; 2 Department of Paediatrics and Child Health, University of Cape Town, Cape Town, South Africa

**Keywords:** respiratory tract infection, paediatrics, respiratory therapy, Southern Africa, chest physiotherapy

## Abstract

**Background:**

Little is known about the prescription, frequency and nature of airway clearance therapy (ACT) in children hospitalised with lower respiratory tract infections (LRTIs).

**Objectives:**

To describe the characteristics and outcomes of children hospitalised with LRTIs at a tertiary paediatric hospital in South Africa and to investigate the role and impact of ACT in these children.

**Method:**

A retrospective folder review of children hospitalised with LRTI between January and June 2015 was conducted, extracting data on demographic characteristics, health condition, ACT interventions and patient outcomes.

**Results:**

A total of 1208 individual cases (median [IQR] age 7.6 (2.8–19.0) months), in 1440 hospitalisations, were included. The majority of children were hospitalised primarily for the management of bronchiolitis. Comorbidities were present in 52.6% of patients during at least one of their hospitalisations. Airway clearance therapy was administered in 5.9% (*n* = 85) of admissions, most commonly conventional (manual) ACT. Transient oxyhaemoglobin desaturation was reported in six children, and one child developed lobar collapse an hour post-treatment. No other adverse events were reported. The median (IQR) duration of hospitalisation was 2.3 (1.5–5.0) days, and the overall mortality rate was 0.7%. Children hospitalised for presumed nosocomial infections and pneumonia had the longest length of stay, were more likely to receive ACT and had the highest mortality rate.

**Conclusion:**

Airway clearance therapy was infrequently used in this population and was more commonly applied in those with nosocomial LRTI and pneumonia.

**Clinical implications:**

Although ACT was generally well tolerated, safety has not been ascertained, and oxygen saturation should be carefully monitored during therapy.

## Introduction

Lower respiratory tract infections (LRTIs), particularly pneumonia, are among the leading causes of mortality in children under 5 years of age worldwide (Liu et al. 2015:430–440). Respiratory disease may cause increased volume and viscosity of pulmonary secretions, ciliary dyskinaesia and ineffective cough, which may impair pulmonary secretion clearance with subsequent sequelae related to airway obstruction (Fink 2007:1210–1221). Physiotherapists can facilitate airway clearance using techniques that mobilise pulmonary secretions, known as airway clearance therapy (ACT) (Morrow 2019:1–12). These techniques can be passive techniques, performed manually by the therapist (e.g. positioning, percussions and vibrations), or active techniques requiring cooperation (e.g. the active cycle of breathing technique, autogenic drainage and positive expiratory pressure techniques) (Morrow 2019:1–12). Although ACT may be used in the clinical management of children with LRTIs, there is a lack of evidence regarding the safety and effectiveness of ACT in this population. In children hospitalised with pneumonia, contradicting results have been found regarding the effectiveness of ACT in this population (Abdelbasset & Elnegamy 2015:219–226; Corten et al. 2018:e1690; Corten, Jelsma & Morrow 2015:256; Lukrafka, Fuchs & Fischer 2012:967–971; Moura Da Silva et al. 2015:eS1052–eS1053; Paludo et al. 2008:791–794). In children hospitalised with bronchiolitis, however, it is agreed that ACT should not be routinely applied (Caffrey Osvald & Clarke 2015:1–3; Figuls et al. 2016:CD004873). A recently published state-of-the-art review concluded that ACT should not be performed routinely in children with LRTI but rather treatment should be prescribed on an individualised basis following clinical assessment (Morrow 2019:1–12).

There is little recent evidence available on current physiotherapy practices relating to the prescription, frequency and nature of ACT in children hospitalised with LRTIs, or investigating the adherence to guidelines. Furthermore, published data regarding adverse events associated with ACT in this context are also lacking.

This study aimed to describe the characteristics, health condition, course and outcome of children hospitalised with LRTIs at a tertiary paediatric hospital, with specific reference to the role and impact of ACT in these children.

## Materials and methods

This was a retrospective folder review of routinely collected clinical data of children hospitalised at Red Cross War Memorial Children’s Hospital (RCWMCH), Cape Town, South Africa, from January 2015 to June 2015, with a clinical diagnosis of any LRTI.

### Participants

Children from birth to 18 years, admitted to any ward in RCWMCH, with any community- or hospital-acquired LRTI were eligible for inclusion. Folders were identified using primary diagnostic codes (ICD 10 codes) for a range of respiratory conditions or clinical signs of respiratory disease, and physiotherapy department records were used to identify patients who were referred for and/or received ACT during the study period.

### Instrumentation

A standard pre-structured data extraction form, validated for content by two experts, was used to record data on demographic information, comorbid conditions, ACTs administered and any associated complications, and patient outcomes. No identifying information, except for folder number, was recorded during data extraction.

For cases with multiple admissions during the study period, demographic data were collected for the first admission only to avoid pseudo-replication.

### Data analysis

Data were analysed using Statistica Version 13 (Statsoft Inc, USA). Data were tested for normality using the Lilliefors test. All numerical data are presented as either mean (standard deviation [SD]) or median (interquartile range [IQR]), and proportions are presented as percentages. A Chi-squared test (with Yates test where appropriate) was performed to test for associations between categorical variables. The influence of all relevant variables on patient outcome measures was analysed using forward stepwise multiple regression analysis. A one-way ANOVA was performed to compare the length of hospitalisation amongst diagnostic groups.

### Ethical considerations

Institutional ethical approval (HREC 717/2015) was obtained from the Human Research Ethics Committee (University of Cape Town) for the study. The need for written informed consent was waived owing to the retrospective study design.

## Results

**FIGURE 1:**
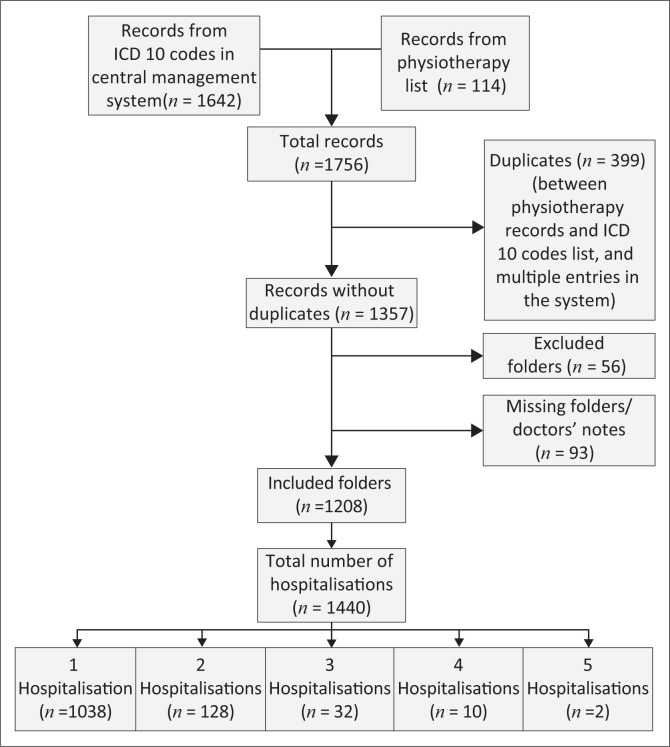
Folder review flow chart.

In total, 1756 cases were initially identified, of which 399 folders were duplicates and 1357 folders were screened for eligibility (Figure 1). Fifty-four folders were excluded as the child did not have an LRTI, and 93 folders were missing. Therefore, a total of 1208 patient folders were included in the descriptive folder review. Of these, 1038 patients (85.9%) were hospitalised once between January and June 2015. The remaining 172 (14.2%) were hospitalised multiple times during the study period.

### Demographic characteristics

The demographic characteristics of the included children can be found in Table 1.

**TABLE 1:** Demographic characteristics of the included children.

Characteristic	Outcome	
	*n*	%
Age (months), median (IQR)	7.6	2.8–19.0
Gender, male (*n*)	720	59.6
Gestation, term ≥ 37 weeks gestational age (*n*)	924	64.2
HIV status (*n*)	-	-
Positive	35	2.9
Negative	937	77.6
Exposed	112	9.3
Unknown	124	10.3

Of the 35 HIV-infected children, 29 (82.9%) had received antiretroviral therapy prior to or at the time of admission. No information was available regarding antiretroviral therapy for the other six patients.

### Health condition

Most hospitalisations were primarily for the management of bronchiolitis (46.0%), followed by pneumonia (36.5%), unspecified acute LRTI (10.0%) and other conditions (0.2%). No primary diagnosis of LRTI was documented in 106 (7.4%) cases; however, the discharge diagnosis included LRTI. After extensively reviewing the clinical course, these children were presumed to have developed nosocomial LRTI during their hospital stay.

Positive sputum or blood cultures were available in 245 admissions (17.0%). Table 2 presents the prevalence of associated organisms, for each diagnostic category, as identified on sputum or blood culture. One child in the category ‘other’ tested positive for respiratory syncytial virus (RSV) and cytomegalovirus.

**TABLE 2:** Prevalence of viral and bacterial organisms, based on*n* = 245 sputum/blood tests (multiple responses per test possible).

Category	Organism	Total		Bronchiolitis, *n*	Pneumonia, *n*	LRTI, *n*	Presumed nosocomial, *n*
		*n*	%				
**Viral**	RSV	108	44.1	27	57	10	13
	Rhinovirus	92	38.0	18	51	7	17
	Adenovirus	79	32.2	19	36	11	12
	Boca virus	32	13.1	9	16	4	3
	Parainfluenza	25	10.2	5	14	5	1
	Influenza	19	7.8	4	7	4	4
	Enterovirus	17	6.9	2	13	1	1
	Human corona virus	15	6.1	1	10	0	4
	Cytomegalovirus	11	4.5	1	7	0	2
	Human metapneumovirus	3	1.2	0	2	0	1
	Parvovirus	1	0.4	1	0	0	0
	**Total viral organisms**	**402**	**-**	**87**	**213**	**42**	**58**
**Bacterial**	*Mycobacterium tuberculosis*	12	4.9	1	7	1	3
	*Klebsiella pneumonia*	11	4.5	1	4	1	5
	*Pseudomonas aeruginosa*	9	3.7	0	2	0	7
	*Haemophilus influenzae*	8	3.3	1	0	1	6
	*Staphylococcus aureus*	6	2.4	0	1	0	5
	*Streptococcus pneumoniae*	4	1.6	0	1	0	3
	PJP	4	1.6	0	4	0	0
	*Acinetobacter baumannii*	3	1.2	0	1	0	2
	*Escherichia coli*	3	1.2	0	2	0	1
	Methicillin-resistant *Staphylococcus aureus*	2	0.8	0	1	0	1
	Moraxella	2	0.8	0	1	0	1
	*Enterobacter cloacae*	2	0.8	0	0	0	2
	*Serratia marcescens*	1	0.4	1	0	0	0
	*Stenotrophomonas maltophilia*	1	0.4	0	0	0	1
	Unspecified gram-positive bacteria	10	4.1	0	7	0	3
	**Total bacterial organisms**	**78**	**-**	**4**	**31**	**3**	**40**
**Total**		**480**	**-**	**91**	**244**	**45**	**98**

RSV, respiratory syncytial virus; PJP, *Pneumocystis jirovecii* pneumonia; LRTI, lower respiratory tract infection.

The majority of children presented with tachypnoea and tachycardia on admission. Signs of respiratory distress were evident in 1361 hospitalisations (94.5%), with tachypnoea being the most common (81.6% of hospitalisations), followed by recessions (69.2%). Other signs of distress were alar flaring (23.5%), tracheal tug (8.5%), head bobbing (4.8%), cyanosis (0.4%) and feeding problems (0.4%). The median (IQR) temperature and peripheral oxygen saturation in room air at admission were 37.0°C (36.7°C – 38.0°C) and 96.0% (94.0% – 98.0%), respectively.

Children received non-invasive and/or invasive mechanical ventilation during 258 hospitalisations (17.9%) (one-hundred and fifty children were diagnosed with pneumonia, 51 were diagnosed with bronchiolitis, 40 were diagnosed with presumed nosocomial LRTI and 17 were diagnosed with unspecified LRTI). Continuous positive airway pressure was the most common form of ventilation (*n* = 234, 90.7%), followed by invasive intermittent positive pressure ventilation (*n* = 62, 24.0%), pressure control ventilation (*n* = 12, 4.7%), high-frequency oscillatory ventilation (*n* = 11, 4.3%), bilevel positive airway pressure (*n* = 11, 4.3%) and synchronised intermittent mechanical ventilation (*n* = 3, 1.2%).

The median (IQR) duration of ventilator support was 3.0 (2.0–5.0) days.

Twenty-five children (2.1%) had a known history of tuberculosis (TB). The episode of TB occurred at a median (IQR) of 8.2 (6.3–45.7) months prior to the first day of admission.

Most children (82.1%) had not been hospitalised for a prior respiratory disease before their first admission during the study period. For those who were hospitalised previously, the most recent hospitalisations occurred at a median (IQR) of 8.9 (4.8–14.6) months prior to the index admission.

Of the total sample, 52.6% had one or more clinically significant comorbidity(ies) during at least one of the hospitalisations in the study period. Respiratory comorbidities were most common (17.6%), followed by cardiac (16.4%) and metabolic/nutritional comorbidities (13.9%). The common chronic and acute comorbidities (present in 10 or more children) are presented in Table 3.

**TABLE 3:** Most common chronic (based on *n* = 1208 children) and acute (*n* = 1440 hospitalisations) comorbidities seen in children hospitalised with a lower respiratory tract infection.

Disease category	Acute or chronic	Comorbidity	*n*	Percentage
**Respiratory**	**Chronic**	**Asthma**		
		Proven	49	4.1
		Suspected	7	0.6
		Upper airway obstruction	27	2.1
		Chronic lung disease, not further specified	11	0.9
	**Acute**	Upper respiratory tract infection (URTI)	47	3.3
		Allergic rhinitis	16	1.1
		Apnoea	16	1.1
		**Pertussis**		
		Proven	13	0.9
		Suspected	2	0.1
		Bronchospasm	11	0.8
**Cardiovascular**	**Chronic**	**Patent ductus arteriosus**		
		Proven	23	1.9
		Suspected	1	0.1
		Ventricular septal defect	22	1.8
		Atrioventricular septal defect	15	1.2
	**Acute**	Anaemia	107	7.4
		Sepsis	72	5.0
**Neurological**	**Chronic**	Seizures or epilepsy	35	2.9
		Cerebral palsy (CP)	23	1.9
		Neuromuscular disorder (NMD)	13	1.1
	**Acute**	Meningitis	10	0.7
**Genetic disorder**	**Chronic**	Trisomy 21	19	1.6
**Musculoskeletal**	**Chronic**	Scoliosis	11	0.9
**Gastrointestinal**	**Chronic**	Gastro-oesophageal reflux disorder (GORD)	51	4.2
	**Acute**	Acute gastroenteritis	91	6.3
**Dermatological**	**Chronic**	Eczema	33	2.7
	**Acute**	Dermatitis	18	1.3
		Candidiasis/fungal rash	11	0.8
**Ear, nose and throat (ENT)**	**Acute**	Otitis media	24	1.7
**Metabolic/nutritional**	**Acute**	Failure to thrive	151	10.5
		Hypothyroidism	10	0.7
**Ophthalmology**	**Acute**	Conjunctivitis	18	1.3
**Other**	**Chronic**	Developmental delay	45	3.7
		Neonatal jaundice	34	2.8
		Dysmorphic features	14	1.2
		Maternal drug abuse during pregnancy or exposure of the child to toxic fumes caused by drugs	10	0.8

A significant association was seen between the primary diagnostic category and the presence of at least one chronic comorbidity (Yates *X*^2^ = 170.5, *p* < 0.001) or acute comorbidity (Yates *X*^2^ = 55.7, *p* < 0.001), with the highest proportion of comorbidities seen in children admitted with pneumonia.

### Airway clearance therapy

In total, 108 cases were referred to the physiotherapy department (7.5%). Airway clearance therapy was given in 85 of these cases (5.9%) (forty-seven cases were diagnosed with presumed nosocomial LRTI, 22 were diagnosed with pneumonia, 14 were diagnosed with bronchiolitis and two were diagnosed with LRTI), with the majority of ACT interventions (75.9%) started during the first week of hospitalisation. Approximately, half the patients received bidaily treatment (49.4%), one received more than bidaily ACT (1.2%) and the remainder (47.1%) received daily ACT. Airway clearance therapies were performed for a median (IQR) of 3.0 (1.0–6.0) days per admission. Table 4 presents an overview of the ACT modalities performed.

**TABLE 4:** Frequency of performed airway clearance therapies in the 83 cases receiving airway clearance therapies (multiple airway clearance therapies could be used per case).

Treatment modality	*n*	Percentage
Vibrations	69	81.2
MPD	46	54.1
Percussions/clapping	32	37.6
Deep breathing exercises	21	24.7
Active gross motor exercises	17	20.0
ACBT	11	12.9
Thoracic compressions	7	8.2
FET/Huff	6	7.1
Bubble PEP	6	7.1
AAD	5	5.9
Oscillating PEP	3	3.5
Blowing bubbles	3	3.5
Chest wall shaking	1	1.2
PEP	0	0.0
AD	0	0.0
**Total**	**227**	**-**

MPD, modified postural drainage; ACBT, active cycle of breathing; FET, forced expiratory technique; PEP, positive expiratory pressure; AAD, assisted autogenic drainage; AD, autogenic drainage.

Transient desaturation occurred in six cases (7.2%) (three desaturated to levels between 85% and 89%, and three desaturated to below 85%), during or immediately after ACT: during positioning in left side lying (*n* = 2), suctioning (*n* = 1), suctioning and vibrations (*n* = 1), percussions and vibrations (*n* = 1) and breathing exercises in the sitting position (*n* = 1). One child presented with right upper lobe collapse more than an hour after ACT. No other adverse events associated with ACT were reported.

A significant association was found between the primary diagnostic category and whether or not ACT was given (Yates *χ*² = 312.5, *p* < 0.001). Airway clearance therapies were most often given for children hospitalised with presumed nosocomial infections and pneumonia, and least often in children admitted with bronchiolitis. Furthermore, children with bacterial organisms were more likely to receive ACT (Yates *χ*² = 158.3, *p* < 0.001).

The most predictive factors for receiving ACT were chronic respiratory comorbidities, neuromuscular disorders, history of previous hospitalisation for a respiratory condition and receiving mechanical ventilation during admission (50.0% of the ventilated patients received ACT). This final logistic regression model provided the best prediction with a -2 Log likelihood ratio of 514.1 (*p* < 0.001).

### Patient outcomes

The median (IQR) length of hospitalisation was 2.3 (1.5–5.0) days, with a significant difference in the mean days of hospitalisation amongst the different diagnostic groups (*p* < 0.001). A post-hoc Tukey’s test identified a significant longer duration of hospitalisation for children with presumed nosocomial infection (mean [SD] 14.3 [18.5] days), and children hospitalised for bronchiolitis had a shorter hospital stay (mean [SD] 2.9 [4.0] days) than children in the other diagnostic categories (pneumonia and unspecified LRTI mean [SD], 6.0 [8.9] days and 5.4 [12.4] days, respectively).

Forward stepwise multiple regression analysis was performed to identify factors that influenced the duration of hospitalisation. The final model accounted for 47.1% of the variance. Mechanical ventilation was the factor that influenced the length of hospital stay the most, explaining 19.4% of variance. It is also seen that children who received ACT stayed in hospital for longer (explaining 6.5% of the variance). Furthermore, chronic comorbidities were more predictive of a longer hospital stay than acute comorbidities (Table 5).

**TABLE 5:** Multiple regression outcomes for duration of hospitalisation.

Step	Predicting factors	*R^2^*	Change in *R^2^*
1	Mechanical ventilation	0.194	0.194
2	+ chronic heart disease	0.332	0.138
3	+ airway clearance therapy (ACT)	0.397	0.065
4	+ oxygen saturation on admission	0.429	0.032
5	+ genetic disease	0.451	0.022
6	+ cerebral palsy	0.462	0.011
7	+ acute comorbidities	0.471	0.008

In total, 10 children (0.7%) died while hospitalised during the study period (Table 6). A significant association between mortality rate and diagnostic category was found (Yates *χ*^2^ = 45.5, *p* < 0.001). Mortality was higher for children who developed presumed nosocomial infections, followed by pneumonia, compared to other diagnostic categories. There was a significant association between delivery of ACT and mortality (Yates *χ*² = 41.96; *p* < 0.001).

**TABLE 6:** Description of the deceased patients.

Participant	Primary diagnosis	Age at time of death	Comorbidities
1	Pneumonia	16.5 years	Liver transplant in 2008: chronic liver reject, sepsis, anaemia, acute gastroenteritis
2	Presumed nosocomial LRTI	12.4 years	Congenital muscular dystrophy, kyphoscoliosis, GORD, developmental delay
3	LRTI	10.9 years	CP (spastic quadriplegia), seizures, scoliosis, microcephaly, cortical blindness, GORD, upper airway obstruction
4	Presumed nosocomial LRTI	7.5 years	CP (spastic quadriplegia), congenital dandy-walker malformation, hydrocephalus, seizures, developmental delay, anaemia, sepsis
5	Pneumonia	5.0 years	Trisomy 21, multiorgan failure, seizures, upper airway obstruction, GORD, history of rickets
6	Presumed nosocomial LRTI	4.5 years	Traumatic brain injury because of pedestrian-vehicle-accident, brain stem death
7	Pneumonia	1.8 years	Pulmonary haemorrhage, bronchospasm, acute gastroenteritis, sepsis
8	Presumed nosocomial LRTI	3.8 months	SMA I, failure to thrive, acute gastroenteritis
9	Presumed nosocomial LRTI	2.8 months	Trisomy 21, atrioventricular septal defect, chylothorax, developmental delay
10	Presumed nosocomial LRTI	1.9 months	Trisomy 13, dysmorphic, polydactyly, bilateral cataract, URTI, failure to thrive, atrioventricular septal defect

GORD, gastro-oesophageal reflux disorder; LRTI, lower respiratory tract infection; CP, cerebral palsy; SMA, spinal muscular atrophy; URTI, upper respiratory tract infection.

## Discussion

The majority of children admitted with LRTIs were younger than 1 year of age, male, HIV uninfected, and born at term, similar to other published studies (Forster et al. 2004:709–716; Hasan et al. 2014:e45–e52; Hatipoglu et al. 2011:508–516; Wolf et al. 2006:320–324). The disproportionate gender representation may relate to differences in sex hormones, influencing immune response through lymphocyte and macrophage function (Muenchhoff & Goulder 2014:S120–126). Furthermore, male infants have narrower peripheral airways compared to female infants, possibly contributing to increased LRTI severity (Tepper et al. 1986:513–519).

In our study, the primary diagnosis of bronchiolitis and pneumonia was made in 46.0% and 36.5% of cases, respectively. This is similar to previous studies that reported between 55% and 60% of in-patients as having bronchiolitis and 35% – 45% diagnosed with pneumonia (Forster et al. 2004:709–716; Hatipoglu et al. 2011:508–516). More viral than bacterial organisms were isolated. This confirms the results of a previous South African study, where human rhinovirus, RSV and adenovirus were most often identified in children with LRTI (White et al. 2016:443–445). Hasan et al. (2014:e45–e52) reported that RSV was the most common viral agent, followed by rhinovirus. This is the same as our results, albeit in different proportions (19.5% and 18.7%, respectively, in Hasan et al.’s report compared to 44.1% and 38.0% here). A study conducted on Turkish children with viral LRTI also reported RSV as the main pathogen (55.6%) in children younger than 1 year of age, followed by parainfluenza, which was the most common viral isolate in older children (Hatipoglu et al. 2011:508–516). We found *Mycobacterium tuberculosis* to be the most common bacterial organism (4.9%), followed by *Klebsiella pneumoniae* (4.5%). Another South African study reported *Acinetobacter baumannii* as the most common bacterial organism, followed by *Klebsiella pneumoniae* (20.6%) (Ghani et al. 2012:e275–e281). Although our rate for *Klebsiella pneumoniae* identification was similar to that reported by Hasan et al. (2014:e45–e52) (4.1%), it was only the seventh most common bacterial agent in the latter study. Our study is limited by the low rate of available sputum/blood cultures, with no standardisation of sampling, potentially overestimating the proportions of some organisms whilst missing others.

Ventilator support was given during 17.9% of the hospitalisations. This proportion is higher than a previous report, where the proportion of children receiving mechanical ventilation was only 1.0% (Wolf et al. 2006:320–324). However, the study did not specify whether non-invasive ventilation was included, in addition to invasive mechanical ventilation (Wolf et al. 2006:320–324).

One or more comorbidities were present in just over half the cases, with respiratory problems the most common, followed by cardiovascular disorders. In a study by Wang, Law and Stephens (1995:212–219) on Canadian children hospitalised with RSV LRTI, the proportion of comorbidities (22.6%) was lower than here (52.6%). This might relate to different methodologies and comorbidity selection (Wang et al. 1995:212–219). Furthermore, we included any LRTI, not merely RSV LRTI.

Of the 5.9% of hospitalisations that received ACT, the majority of treatments were commenced in the first week of hospitalisation. However, seven children only received ACT more than 2 weeks after admission. Vibrations and modified postural drainage (excluding the head-down position) were performed most often, with 49.4% of the children receiving bidaily treatment. Airway clearance therapy is not recommended as routine management for children with bronchiolitis, by the American Association for Paediatrics, based on the results found in a systematic review by Figuls et al. (American Academy of Pediatrics 2014:e1474–e1502; Figuls et al. 2016:CD004873). Although the National Institute for Health and Care Excellence agrees with this recommendation for most children, they do specify that ACT can be given to children with bronchiolitis with relevant comorbidities, if requiring ACT for facilitation of mucus clearance (Caffrey Osvald & Clarke 2015:1–3). In our study, ACT was given less frequently to children with bronchiolitis or unspecified LRTI, which conforms to the guidelines for the management of children with bronchiolitis.

In children with pneumonia, little evidence is available regarding the use of ACT as part of disease management. Two systematic reviews have been published, including up to three randomised controlled trials on this topic, without clear recommendations for or against the use of ACT in these children (Chaves et al. 2013:CD010277; Corten et al. 2015:256). However, small benefits have been reported in other studies (Abdelbasset & Elnegamy 2015:219–226; Santos et al. 2009:23), indicating the need for further research in this field. The children who were most likely to receive ACT were those with bacterial nosocomial infections or pneumonia and those with chronic comorbidities. Therefore, it is recommended that further research be conducted on the use and safety of ACT in children presenting with these conditions.

Adverse events of ACT are rarely mentioned and described in the literature, which was also the case in our study. Six children did, however, desaturate during ACT. One child presented with a lobar collapse more than one hour after ACT, and given the time delay, it is unlikely that this adverse event was directly related to ACT. Owing to the observation of desaturation during ACT, monitoring of peripheral oxygen saturation is recommended during the performance of ACT. A study on the use of ACT in children with bronchiolitis mentioned that no adverse events occurred; however, it is unclear as to which adverse events were under consideration (Postiaux et al. 2011:989–994). As the rate of adverse events was low in our study and very little literature is available on this topic, ACT appears to be safe for use in children with LRTI, but this requires confirmation in prospective clinical studies.

Chronic comorbidities were the most likely predictors for receiving ACT during hospitalisation. Children with presumed nosocomial infections were more likely to receive ACT, followed by children hospitalised for pneumonia. These data, however, may have been biased because the majority of children classified as presumed nosocomial infections were included based on physiotherapy referral and not through diagnostic code search identification. In children hospitalised for pneumonia, multiple comorbidities were identified, for which ACT might be indicated. Children with positive bacterial culture were treated more often by the physiotherapists than children with viral isolates. No previous studies have investigated the association between ACT and isolated organisms; therefore, confirmation of the results in a larger prospective study is recommended.

Children were hospitalised for a median of 2.3 days; however, children with presumed nosocomial infections were hospitalised for a significantly longer duration (14.3 days). In addition to clinical outcomes, cost prevention and high demands for hospital beds in lower-resourced countries could be contributing factors for short duration of hospital stay (Argent et al. 2014:7–14). However, three randomised controlled trials on children hospitalised with pneumonia reported a median duration of hospital stay of six to eight days (Corten et al 2018:e1690; Lukrafka et al. 2012:967–971; Paludo et al. 2008:791–794), which is similar to the duration of hospitalisation for pneumonia in our study.

The length of hospital stay was associated with whether or not children received ventilator support; had chronic heart conditions; received ACT, desaturation on admission; and had cerebral palsy, genetic disorder or acute comorbidities. In a study conducted by Rodriguez et al. (2014:269–276), predictors for disease severity in children with RSV LRTI, partially based on length of hospital stay, were aged younger than 6 months, born prematurely, with a pre-existing lung disease or congenital cardiac disease. Our finding that receiving ACT was associated with increased duration of hospital stay may reflect the increased likelihood of receiving ACT in those who developed nosocomial infections (Green et al. 2015:305–312).

However, the RCT by Lukrafka et al. also reported a median 2 days longer hospital stay in children who received ACT, compared to controls, which was not statistically significant, possibly owing to insufficient sample size (Lukrafka et al. 2012:967–971). Other studies have not reported significant differences for length of stay when receiving ACT in children hospitalised with pneumonia (Corten et al. 2018:e1690; Paludo et al. 2008:791–794). Prospective studies are recommended to confirm these results and to determine causality.

Pneumonia is still the most common cause for mortality in children younger than 5 years of age worldwide (Liu et al. 2015:430–440). In our study, the overall mortality rate for children with an LRTI was low; however, a greater proportion of children with presumed nosocomial infection (5.7%) died. The mortality rate for children with a clinical diagnosis of pneumonia was 0.6%. These mortality rates, although slightly higher, are comparable to the rates found in children younger than 5 years hospitalised with acute LRTI in rural Thailand, with 0.3% overall mortality rate (Hasan et al. 2014:e45–e52). All children included in our study, who died during the study period, presented with multiple comorbidities and seven of the 10 children were older than 4 years of age. The cause of death could therefore be multifactorial and not solely attributed to LRTI. Airway clearance therapy was associated with an increased mortality rate, which has not been previously reported. However, causality cannot be determined on the basis of this study design. Considering most children admitted with pneumonia had comorbidities, this may explain the higher mortality observed in this group.

## Conclusion

This study revealed that a relatively small proportion of children with LRTI received ACT. Airway clearance therapy was mostly applied in children with presumed nosocomial infections, followed by pneumonia. Given the paucity of high-level evidence, ACT is therefore used in clinical practice based on physicians’ and physiotherapists’ expert opinion. It is therefore recommended that more research regarding ACT in children with nosocomial infections and pneumonia be conducted. This is particularly important considering that the duration of hospitalisation was longer and the mortality rate was higher in children with nosocomial infections and pneumonia, compared to those admitted with other LRTIs, especially bronchiolitis.

This study also found that ACT, as performed at this research site, appears relatively safe to perform in children with LRTI. However, the study was not designed or powered to determine safety, and further prospective, controlled clinical trials are recommended to confirm this finding. It is recommended that peripheral oxygen saturation be continuously monitored during ACT, in order to promptly identify desaturation and implement appropriate management.

As more than half the children included in this study presented with comorbidities, research is warranted to investigate the use of ACT in children hospitalised with LRTI and comorbidities, both chronic and acute.
